# Neutrophil-Mediated Progression of Mild Cognitive Impairment to Dementia

**DOI:** 10.3390/ijms241914795

**Published:** 2023-09-30

**Authors:** KyoungJoo Cho

**Affiliations:** Department of Life Science, Kyonggi University, Suwon 16227, Republic of Korea; kcho0611@kgu.ac.kr; Tel.: +82-31-249-1365

**Keywords:** mild cognitive impairment, neutrophil, dementia

## Abstract

Cognitive impairment is a serious condition that begins with amnesia and progresses to cognitive decline, behavioral dysfunction, and neuropsychiatric impairment. In the final stage, dysphagia and incontinence occur. There are numerous studies and developed drugs for cognitive dysfunction in neurodegenerative diseases, such as Alzheimer’s disease (AD); however, their clinical effectiveness remains equivocal. To date, attempts have been made to overcome cognitive dysfunction and understand and delay the aging processes that lead to degenerative and chronic diseases. Cognitive dysfunction is involved in aging and the disruption of inflammation and innate immunity. Recent reports have indicated that the innate immune system is prevalent in patients with AD, and that peripheral neutrophil markers can predict a decline in executive function in patients with mild cognitive impairment (MCI). Furthermore, altered levels of pro-inflammatory interleukins have been reported in MCI, which have been suggested to play a role in the peripheral immune system during the process from early MCI to dementia. Neutrophils are the first responders of the innate immune system. Neutrophils eliminate harmful cellular debris via phagocytosis, secrete inflammatory factors to activate host defense systems, stimulate cytokine production, kill pathogens, and regulate extracellular proteases and inhibitors. This review investigated and summarized the regulation of neutrophil function during cognitive impairment caused by various degenerative diseases. In addition, this work elucidates the cellular mechanism of neutrophils in cognitive impairment and what is currently known about the effects of activated neutrophils on cognitive decline.

## 1. Introduction

Aging is a biological process accompanied by many molecular changes, resulting in 31 alterations of the cellular environment, thereby creating an inflammatory condition [1]. For this reason, aging is the primary risk factor in neurodegenerative diseases and cardiovascular diseases such as atherosclerosis and stroke. Aging is the accumulation of reactive oxygen species (ROS) that cause cellular component damage, such as mitochondrial dysfunction [2] and chronic neuroinflammatory condition [3]. In the brain, aging affects synapse density and synaptic function, resulting in reduced synaptic transmission and plasticity [4]. The aged brain has altered structure and cognitive function that leads to brain atrophy, particularly in the hippocampus and prefrontal cortex [5]. Cognitive impairment is also known as dementia and is characterized by a decline in memory, thinking, perception, language, decision-making, planning, and reasoning [6]. Cognitive function can be categorized into specific cognitive domains such as processing speed, attention, memory, language, visuospatial abilities, and executive functioning and reasoning [7]. In the case of Alzheimer’s disease (AD), the National Institute on Aging and the Alzheimer’s Association provided guidelines defining dementia as comprising three phases: preclinical AD (early pathologic changes in the brain of cognitively normal individuals), MCI (symptomatic early dementia), and dementia.

Mild cognitive impairment (MCI) describes individuals who have some cognitive deficits without dementia. The MCI can progress and lead to severe cognitive dysfunction in the aged brain. Approximately 20% of patients with MCI progressed to further cognitive deficits and dementia over time [8]. During the transition from MCI to dementia, the main causes and risk factors have been identified as non-modifiable factors, such as aging and genetic characteristics, and modifiable factors, such as physiological conditions due to degenerative disease and clinical characteristics [9]. Recent studies have emphasized the importance of the innate and adaptive immune systems in the pathophysiology of age-related diseases [10]. Lymphocyte levels are significantly reduced in patients with MCI and AD compared with those in healthy controls [11,12]. Decreased interleukin (IL)-10, IL-4, IL-2, and IL-1β levels results in a dysregulated peripheral immune system that worsens disease progress in the early stage of dementia with Lewy bodies and MCI-AD [12]. Patients with dementia, including AD, exhibit prevalent activation of the innate immune system. Recent studies have shown that changes in peripheral neutrophils in individuals with MCI can be a marker for predicting a decline in executive cognitive function in cerebral small-vessel disease [13]. The findings provide an exploration point for the use of neutrophils as prediction markers in the progression or prognosis of MCI to dementia, including AD. In addition, the neutrophil-to-lymphocyte ratio correlates with amyloid burden and cognitive decline in patients with AD [14,15]. This review provides information on how neutrophils function and are involved in age-related cognitive impairment and progression from MCI to severe dementia.

## 2. Cognitive Function, Decline, Impairment, and Dementia

Cognitive dysfunction, including AD, is an irreversible degenerative brain disorder. AD is the most common form of dementia, comprising approximately 60–80% of dementia cases [14]. The incidence and prevalence of AD are increasing with the growth of the older adult population and extended lifespans in the current society. Normal cognitive function changes are associated with aging, and in individuals with MCI, it is prone to deteriorate or to progress to dementia. To understand and predict the progression or transition of MCI, biomarkers from cerebrospinal fluid (CSF), positron emission tomography, and magnetic resonance imaging have been used in clinical studies and research approaches during the last decade. Individuals with MCI were identified as a group with a high risk of progression to dementia. Neurodegenerative diseases and cognitive impairment are heterogeneous and diverse. It is important to identify the factors contributing to the progression from MCI to dementia for clinical prognosis, risk stratification, and selection of potential treatments. Considering that predictive biomarkers alone have limitations, various assessments have been conducted using the accepted standard AD biomarkers. This section introduces the recent reports.

### 2.1. Mild Cognitive Impairment

Several definitions share the current concept of MCI to identify patients with pre-AD. The Winblad criteria include individuals with cognitive impairment in domains beyond memory impairment, which are divided into amnestic MCI (aMCI) with memory impairment and non-amnestic MCI (naMCI), which is significant cognitive impairments in domains other than memory. The aMCI criteria are based on a global deterioration scale (GDS) score of 3 points and cognitive impairment—no dementia [16,17]. Recently, there have been developments in defining MCI criteria, with a primary emphasis on categorizing MCI subtypes, resulting in the classification of aMCI, naMCI, single-domain impaired aMCI or naMCI, and multiple-domain impaired aMCI or naMCI [17]. As a clinical assessment, mental status tests are mainly performed with mini-mental state examination (MMSE) and clinical dementia rating (CDR), and the test is determined to be MCI or dementia, using the mean and standard deviation considering age and education level.

A meta-analysis reported the reversion rate was approximately 27.57%, whereas reversion rates were much lower in clinical studies than in community-based studies [18]. This may depend on the study settings, sample size, duration of follow-up, geographic regions, and other patient-based factors. Generally, approximately 20% of patients with MCI experience a spontaneous aggravation of cognitive deficits over time [8]. Many studies have identified novel targets for monitoring disease progression and improving prognosis. Research approaches for biomarkers have been studied to evaluate abnormal CSF levels of amyloid and tau among individuals with normal cognition and patients with MCI or dementia [16,19,20]. A recent study longitudinally collected samples from 269 cognitively healthy individuals and measured the synaptic protein neuronal pentraxin 2 (NPTX2) levels [16,19,20]. NPTX2 plays a role in homeostatic regulation in the CNS, including memory in the brain [21]. The reduced levels of NPTX2 (almost at baseline) shortened MCI onset, which can predict MCI onset time when combined with other AD biomarker levels. According to a long-term follow-up study (7 years) that examined the relationship between NPTX2 levels and MCI onset, however, NPTX2 and AD biomarker changes in CSF Aβ42/Aβ40, p-tau181, or t-tau are independently associated with clinical progression [19]. Since the altered NPTX2 levels precede AD clinical symptoms and NPTX2 is detected with a synaptic degeneration marker rather than an AD-specific marker, the level of NPTX2 may be used as a prediction marker with broad applicability.

### 2.2. Severe Cognitive Impairment (Dementia, AD)

Dementia subtypes are multifactorial because they involve interactions between biological aging, genetic factors, and environmental factors. Therefore, differential degrees of interaction among these factors could influence variations in age of onset, disease duration, neuropathological patterns, and disease progression [22,23]. After the identification of AD-related plaque (amyloid beta) and tangle (tau) pathological conditions, a number of studies have been conducted to understand disease progression, develop disease prognosis, and apply therapeutics to overcome dementia based on the pathology of AD. In these current hypotheses, AD heritability is 60~80%, but little is known about the genetic factors specifically related to MCI-to-AD progression except the effect of the apolipoprotein E (ApoE) ε4 allele [24]. With the inheritance of the ApoE ε4 allele, disease onset in early age is related to dysregulated lipid transport, altered blood–brain barrier (BBB) permeability, and upregulated vascular amyloid deposition [25]. Recent studies reveal that vascular damage contributes to cognitive impairment and dementia, and accounts for 10–20% of dementia cases [26,27,28]. Vascular dementia (VaD) frequently manifests post stroke through strategic and multi-infarction mechanisms involved in excitotoxicity, ROS generation, BBB breakdown, neuroinflammation, and cell death [28,29].

To target cognitive impairments, including MCI, VaD, and AD, basic research and clinical interventions relating to pharmacological and non-pharmacological aspects have been attempted. Cognitive training, stimulation, and rehabilitation are approaches for the prevention and treatment of cognitive decline [30]. Since cognitive impairment is complex and intervention for dementia requires multi-domain interventions, large clinical trials with multi-domain interventions have been reported, such as the Finnish Geriatric Intervention Study to Prevent Cognitive Impairment and Disability [31], Multidomain Alzheimer Preventive Trial (MAPT) [32], and Prevention of Dementia by Intensive Vascular Care [33]. There have been proposed interventions for cognitive impairment, including cholinesterase inhibitors, memantine, antidiabetic agents, probiotics, and cerebrolysin as pharmacological interventions and cognitive training, cognitive stimulation, and cognitive rehabilitation as non-pharmacological interventions. Currently, a variety of new drug targets have been identified to improve cognitive function and delay the progression of cognitive decline. Moreover, new drug candidates are still in development.

## 3. Innate Immunity, Neutrophils, and Neuroinflammation

Neutrophils are the first line of defense against invading pathogens. Neutrophils are thought to be the most important cells for acute inflammatory responses; however, recent data from neutrophil studies indicate an impact on chronic inflammatory processes that contribute to neurological diseases [34,35,36,37]. Neutrophils engulf harmful cellular debris or invade pathogens via phagocytosis, and multiple granules and vesicles release inflammatory factors upon activation [5]. Inflammation is involved in the pathophysiology of neurodegenerative diseases, and multiple inflammatory processes are crucial for the progression of CNS diseases [38]. Systemic inflammation and neutrophils have been implicated as predictors and risk factors of cognitive dysfunction caused by various neuronal diseases. This section introduces the role of inflammation, activated neutrophils in the CNS, and vascular inflammatory conditions in neurodegenerative diseases. Moreover, defense mechanisms against pathogens and the aggravation of cognitive function are discussed.

### 3.1. Innate Immunity and Neuroinflammation

Systemic inflammation can trigger a pro-inflammatory environment in the CNS, and inflammation is increasingly being implicated as a risk factor for VaD. Inflammation is the hallmark of neurodegenerative diseases [39]. In the population aged > 65 years, a high monocyte count has been proposed to be a predictor of premature occurrence of coronary events, although there is a weak relationship between white blood cell count and cardiovascular disease [40]. Aging is a concomitant process that dysregulates immune responses and modulates the relative ratio of immune cell proliferation [41].

Innate and systemic immune responses occur throughout disease progression; consequently, pro-inflammatory cytokines have been detected in both the peripheral [42,43] and central nervous system [44]. Peripheral leukocytes and functional lymphocytes are altered in patients with AD [45]. Locally recruited immune cells are responsible for immune cell dynamics, because they can eventually lead to aberrations in the peripheral immune system [46]. The active peripheral immune response in AD is linked to this response and has been used as a marker for disease diagnosis [47]. Complement factor C1q is the initial component of the innate immunity pathway for phagocytosis and for microglia to remove abnormal protein aggregates [48]. Opsonization and synaptic pruning by C3 play key roles in the presentation of dementia in AD [49]. Genetic or pharmacological interruption of complements C1q, C3, or C5 in innate immune signaling is expected to prevent cognitive impairment caused by synaptic loss [49,50,51].

CNS microglia precede neutrophil activation. Recent genome-wide association studies have reported that specific microglial genes, such as CD33, triggering receptor expressed on myeloid cells 2 (TREM2) and human leukocyte antigen—DR isotype, are associated with the susceptibility to late-onset AD. Several animal models have shown that neutrophil depletion increases pathological disease severity [52]. Neutrophil production is altered with age due to defective regenerative potential. Hematopoietic stem cells (HSCs) of aged mice are changed via myelopoiesis and lymphopoiesis in the bone marrow [53,54]. Cytokines, such as IL-6 and IL-1 β, compromise HSC self-renewal and lead to myeloid bias. Under aged physiological conditions, low levels of cytokines (IL-1β), chemokines (chemokine ligand 5, CCL5), or lipopolysaccharide (LPS) may disturb the balance toward HSCs with myeloid potential [55].

### 3.2. Neutrophil Activation

Blood biomarkers for CNS diseases are required for diagnosis, prognosis, and therapeutic applications [56]. In a clinical report, lymphocyte levels were significantly reduced in patients with AD and MCI compared with those in healthy controls [57]. Inflammatory factors such as IL-10, IL-1β, IL-4, and IL-2 were also changed in patients with cognitive dysfunction, which supports the role of the peripheral immune system early in the cognitive-decline process [57]. Peripheral neutrophil activation indicates a pathological environment and can be detected by blood neutrophil gelatinase-associated lipocalin (NGAL) and myeloperoxidase (MPO) concentrations [58]. Neutrophils are pro-inflammatory indicators and play a critical role as the first responders to inflammation; however, their role has been implicated in chronic diseases. In chronic inflammation, lymphocytes play a major role by secreting interleukin (IL)-10 [59], and the neutrophil/lymphocyte ratio (NLR) has recently emerged as a reliable indicator of systemic inflammatory conditions [60]. Particularly under disturbed inflammatory conditions, increased NLR is an independent risk factor for MCI [11,61] and is also demonstrated in patients with a history of acute ischemic stroke [62] or carotid endarterectomy [63]. The NLR is a clinical marker of peripheral inflammation. Differences in the NLR are driven by risk factors such as age, sex, and ApoEε4 status.

The markers to detect neutrophil function in peripheral blood are tumor necrosis factor (TNF) for neutrophil activation and survival [64,65]; macrophage inflammatory protein-1β and IL-8 for neutrophil trafficking and secretion [66]; NGAL for neutrophil anti-microbial molecule [67,68,69]; and myeloperoxidase (MPO) for neutrophil-related oxidative stress [67]. Several studies have reported that neutrophil-related markers in the peripheral blood can predict the change from cognitive function decline to dementia in patients with MCI [13]. Neutrophils are highly adaptive to the tissue environment. Neutrophils translocate into the brain and colocalize with cerebral blood vessels and amyloid plaques within the brain parenchyma [70]. Recent reports have shown that neutrophil adhesion in the small cerebral vessels may mediate changes in cognition [71]. Neutrophils cause telomere dysfunction and produce ROS from aged non-immune cells. In the cross-talk between neutrophils and senescent cells, C-X-C motif chemokine ligand (CXCL)1 enhances neutrophil reverse trans-endothelial migration in aged mice under inflammatory-conditioned tissues [72]. Neutrophils are generally removed from circulation and cleared by bone marrow macrophages through efferocytosis when aged [72]. As neutrophils age, senescent neutrophils release IL-1β and are difficult to remove from tissue.

### 3.3. Neutrophil Function: NET and NETosis

**Figure 1:**
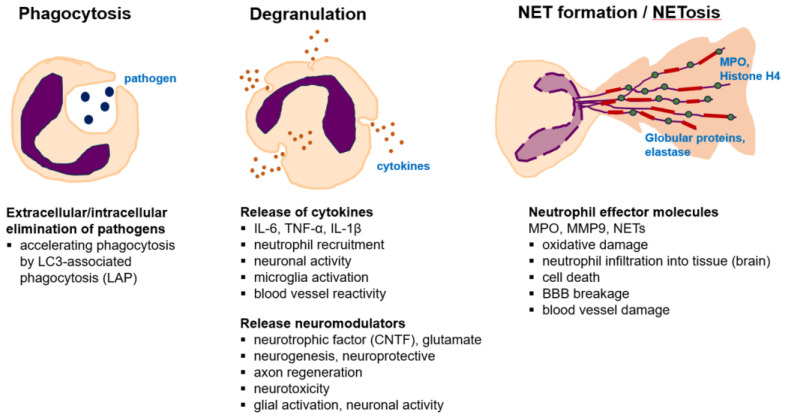
Neutrophil activation and cellular function. Neutrophils are activated under various conditions of cellular damage and react to control it. Neutrophils act in the following ways: phagocytosis, degranulation, and NET formation. IL6, interleukin-6; CNTF, ciliary neurotrophic factor; MPO, myeloperoxidase; NET, neutrophil extracellular trap; BBB, blood–brain barrier; MMP, matrix metalloproteinase.

Neutrophils have a broad spectrum of functions such as phagocytosis of infected microorganisms, release of pro-inflammatory cytokines and chemokines, oxidative stress by overexpressing ROS, and formation of neutrophil extracellular traps (NETs). NETs are web-like structures released by neutrophils that consist of unwound DNA with histones and granular proteins [73] (Figure 1). NETs bind to microorganisms with web-like structures, inhibit their spread, release highly concentrated antimicrobial peptides, and kill bacteria, fungi, and parasites. A crucial step in NET formation is chromatin recondensation mediated by citrullination of histones by peptidyl arginine deiminase 4, which causes nuclear swelling [74]. Cholesterol crystals induce inflammation by activating macrophages and neutrophil inflammasomes in animal models [75]. Inflammasomes are activated by pro-inflammatory cytokines IL-1β and IL-18 [76,77], and activated caspase-1 or caspase-11 cleave gasdermin-D [78]. The induction of NETs by hydrogen peroxide stimulates neutrophils, which can induce NET formation [79]. There are two different pathways of NET formation according to ROS production by nicotinamide adenine dinucleotide phosphate (NADPH) oxidase (Nox): the Nox-dependent pathway and the Nox-independent pathway. Activation of each pathway activates kinases in different ways. Generally, NET formation has been studied as one of the main mechanisms of the antibacterial effect of neutrophils; however, current studies have reported that excessive NET formation is involved in the pathogenesis of various diseases such as atherosclerosis [80], lung injury [81], systemic lupus erythematosus [82], and tumors [83].

In the blood, interactions between neutrophils and activated platelets occur by binding to the p-selectin glycoprotein ligand-1 of neutrophils and the p-selectin of platelets [84], which help neutrophils undergo NET formation [85,86]. Furthermore, platelets adhere to NETs via von Willebrand factor and form platelet aggregates [87,88]; therefore, NETs are involved in thrombosis in vascular diseases [88,89]. NETs observed in intravascular and intraparenchymal sites contribute to BBB damage and neuronal injury in neurodegenerative diseases such as AD [90,91]. Neutrophil-associated brain damage triggers the migration of neutrophils that produce IL-17, which is deleterious to neurons. Damaged neurons and the BBB recruit additional neutrophils, thereby producing a vicious cycle [92,93]. The balance between macrophages and neutrophils mediates their clearance and targets. Ultimately, neutrophils mediate pyroptosis and NETosis [87]. NETosis involves neutrophil lysis to remove NETs, such as necrosis and loss of membrane integrity [94]. The appropriate clearance of neutrophils is critical for maintaining blood and tissue homeostasis. The removal of neutrophils by apoptosis, which is required for the resolution of inflammation, is particularly relevant in injured tissues. Efferocytosis promotes anti-inflammatory cytokine signaling and prevents secondary cellular necrosis [95]. Activated neutrophils and NETosis have been detected in neurodegenerative diseases and may be involved in the progression of MCI to dementia.

The activation of neutrophils in the CNS and their interaction with other immune cells are important factors in the progression of these diseases. Systemic inflammation and neutrophil activation have been identified as predictors and risk factors of cognitive dysfunction caused by neuronal diseases. Blood biomarkers such as NGAL and MPO can be used to detect neutrophil activation and indicate a pathological environment. Understanding the role of neutrophils in CNS diseases including vessel diseases allows for the development of targeted therapies that aim to modulate their function or reduce their detrimental effects. Inhibiting neutrophil activation or avoiding their migration to the brain could potentially reduce inflammation and slow down the progression of neurodegenerative diseases. Additionally, targeting the formation or clearance of NETs could offer therapeutic strategies for managing these diseases. Conclusively, neutrophils’ role in inflammation, their activation in the CNS, and their potential as predictors and risk factors in cognitive dysfunction make them important targets for therapeutic interventions.

## 4. Activated Neutrophil-Mediated Cognitive Impairment

Neutrophil numbers and activation are altered in various neurodegenerative diseases and are potentially involved in degenerative disease progression and disease severity. Neutrophils and activated leukocytes contribute to neuroprotection and restoration. Furthermore, neutrophils have been proposed as a promising target for overcoming neurodegeneration and supporting neuronal regeneration by modulating neutrophil maturation, release, activation, and infiltration. The roles of neutrophils in neurodegenerative diseases are discussed in the following sections.

### 4.1. Atherosclerosis-Derived Cognitive Impairment and Neutrophils

Atherosclerosis is vascular dysfunction caused by lipid accumulation in inner blood vessels. It is characterized by narrowing and hardening of the arteries with plaque that is composed of substances like cholesterol, fatty deposits, calcium, and cellular waste products. The disease is a complex inflammatory disease that results in an immune response in the large and medium-sized arteries [96,97,98,99]. Vascular dysfunction in atherosclerosis results in inflammation, a breakdown of the BBB, and neurovascular dysfunctions, which could impair clearance of Aβ and eventually link to cognitive decline [100]. Cardiovascular and cerebrovascular diseases, including atherosclerosis, are associated with aging, hypertension, hyperlipidemia, and inflammation. All of these conditions could increase the risk of developing AD along with cognitive impairment [57].

**Figure 2:**
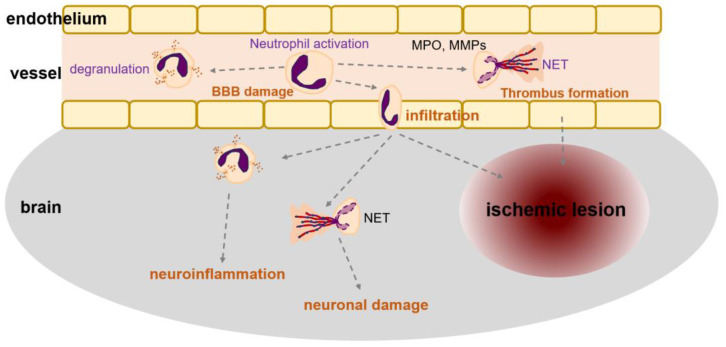
Neutrophils and vessel. Neutrophils are activated when formed thrombus is dysregulated in cardio/cerebral vessels. It might result in atherosclerosis or ischemic stroke. MPO, myeloperoxidase; NET, neutrophil extracellular trap; BBB, blood–brain barrier; MMP, matrix metalloproteinase.

**Figure 3:**
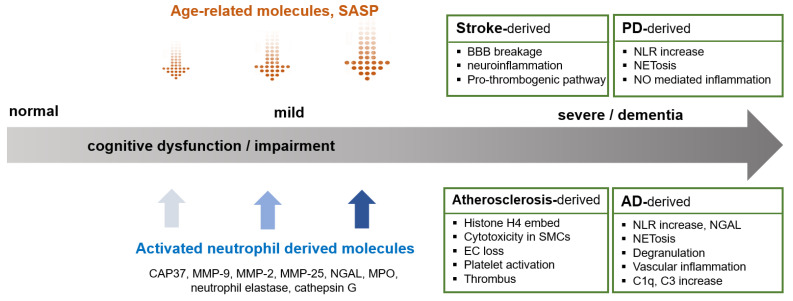
Neutrophils, cognition, and progression of cognitive impairment in neurological disease. Activated neutrophil-derived molecules and aging-related secreting factors are involved in progression of cognitive decline mediated by neurological and neurodegenerative diseases. NLR, neutrophil/lymphocyte ratio; NGAL, neutrophil gelatinase-associated lipocalin; MPO, myeloperoxidase; SASP, senescence-associated secreting protein.

The role of neutrophils in the pathogenesis of atherosclerosis is well established. Although neutrophils respond to acute immune responses, they can play a crucial role in chronic inflammation associated with atherosclerosis [96,101]. Atherogenic processes hamper cholesterol efflux, activate inflammation, disturb the balance between the myeloid and lymphoid hematopoietic stem and progenitor cells, and enhance myelopoiesis. These responses increase the number of blood neutrophils and monocytes [102,103]. Increased neutrophil levels in atherosclerosis can further facilitate neutrophil aggregation in the brain, and activated neutrophils can lead to additional damage in various tissues, including the brain, in a paracrine manner (Figure 2). Neutrophils are also expressed in the vasculature of the brain, and the activated neutrophils may induce cerebrovascular cell apoptosis and dysfunction. The granules in neutrophils contain components for cell lysis including azurocidin, proteinase 3, α-defensin, MPO, LL37 (known as cathelicidin-related antimicrobial peptide in mice), and Cathepsin G. These enzymes activate the endothelium and act as chemoattractants for monocytes [104]. They also regulate endothelial cell permeability by enhancing the expression of adhesion molecules and chemokine receptors in the endothelial cells [104]. When neutrophils arrive at plaques, they release NETs, MPO, and proteases including matrix metalloproteinase-9 (MMP-9). Activated neutrophils overproduce ROS, and increased ROS levels induce endothelial dysfunction and instability in atherogenic plaques [101]. A recent study demonstrated that Histone H4 released by NETs binds and lyses vascular smooth muscle cells (VSMCs), induces apoptosis of VSMCs, and breaks the BBB [105]. Growing evidence for the role of NETs in many CNS diseases has been reported, and neutrophil activity and NETs, including ROS, have been associated with AD progression in clinical studies [13] (Figure 3).

### 4.2. Ischemic Stroke-Derived Cognitive Impairment and Neutrophils

Ischemic stroke occurs because of the migration of local thrombosis or peripheral circulatory clots into the brain and subsequently causes blockage of blood supply to the brain. The cessation of blood flow to the brain leads to the formation of an ischemic core and the surrounding ischemic penumbra, which can be recovered [78]. Ischemic stroke induces activation of brain resident immune cells and infiltrating hematogenous myeloid cells [106,107]. After an ischemic stroke, the inflammatory response is a key element in stroke pathology and is one of the main causes of secondary brain damage [108]. Ischemic injury at the onset of stroke triggers a robust inflammatory response involving blood cell recruitment via cytokines, adhesion molecules, and chemokines [109,110]. Neutrophils move rapidly from the bone marrow and conduct an innate immune response that occurs a few hours after occlusion and reaches maximal levels at early time points of one to three days [109,111]. Neutrophil infiltration in ischemic lesions is an important pathogenic factor [112] (Figure 2).

When neutrophils were depleted in a middle cerebral artery occlusion (MCAO) mouse model, the infarct volume was significantly reduced [113]. Neutrophil depletion reduces BBB damage and improves functional recovery in permanent MCAO (pMCAO). In the other hand, the neutrophils involved in stroke damage are deformed by rosiglitazone (RSG), a neuroprotective reagent, and eliminated by neutrophil apoptosis [114]. This showed that neutrophil disposal is an important step in the resolution of inflammation and restoration and maintenance of tissue homeostasis [115,116]. Neutrophils involved in this response progressed to NETosis [117]. NETosis was detected after 12 h of MCAO and is related to BBB destruction due to neutrophil elastase, by which neutrophils can attack the BBB with decondensed chromatin [118,119].

Neuroinflammation by primary ischemic injury exacerbates ischemic neuronal damage. Neutrophils enter ischemic tissues and play a detrimental role in secondary infarct growth [120,121]. In ischemic stroke, NETs in thrombi were first reported in the presence of citrullinated histones [122]. In studies on neurological dysfunction after ischemic stroke, citrullinated histone H3 and extracellular DNA fibers have been observed in the ischemic brain [118,123]. NETs are also implicated in ischemic stroke pathogenesis via promotion of coagulation and thrombosis as well as atherosclerosis pathogenesis [89,124] (Figure 2). High-mobility group box-1 (HMGB1) is released from neuronal nuclei after acute damage, such as ischemic stroke [117,119]. HMGB1 is a prototypic danger-associated molecular pattern and serves as a mediator in forming NETs. The potential mechanism of NET formation begins with the formation of a ternary complex consisting of DNA, fibrinolytic proteins, and fibrin [125] (Figure 1). Consequently, these procedures impede blood flow in the vessels [125]. In activated neutrophils, HMGB1 is extruded into the extracellular space during NETosis and exacerbates neuroinflammation by recruiting and activating neutrophils and other immune cells [119].

In ischemic conditions of the brain, N2 neutrophils activate neutrophils similar to M1 and M2 macrophages [114,126]. N2 polarization of neutrophils is associated with their ability to undergo phagocytosis. The removal of debris from the inflamed tissue is important for restoring tissue homeostasis and ameliorating stroke outcomes. After completing the role of neutrophils, dead neutrophils disintegrate and release cargo, such as serine proteases or other cationic proteins. This contributes to the inflammatory response and tissue destruction. The polarization of neutrophils to the N2 phase is important for reducing acute inflammation, and phagocytosis of neutrophils promotes the secretion of anti-inflammatory mediators. Subsequently, the brain recovers after stroke [116] (Figure 3).

### 4.3. AD-Derived Cognitive Impairment and Neutrophils

The pathological factors of AD include inflammation, the release of cytotoxic products, neutrophil activation and migration, and increased amounts of ROS [13,14]. Familial AD is caused by genetic mutations in amyloid precursor protein (APP), presenilin 1/2, or subunits of γ-secretase. Familial AD symptoms have an early onset, whereas sporadic AD is caused by genetic mutations combined with environmental risk factors such as aging, tau, p-tau, Aβ overproduction, and mistreatment of abnormal protein aggregates [127]. Several genetic factors for AD have been identified, including Apolipoprotein E (ApoE) and TREM2 [127]. The ApoE is encoded by three common alleles, ε2, ε3, and ε4. Patients with two ε4 copies are vulnerable to AD onset by as much as 12-fold [128,129]. TREM2 is involved in microglial function and inflammation along with other genes such as CD33, complement receptor type 1, adenosine triphosphate-binding cassette sub-family A member 7, and SH-2 containing inositol 5′ polyphosphatase 1 [130,131]. The TREM family comprises cell surface transmembrane glycoproteins consisting of V-immunoglobulin extracellular domains and cytoplasmic tails [132]. The functions of TREM2 have been well characterized over the last decade, and TREM2 accelerates the rate of phagocytosis. When TREM2 was decreased in microglia and macrophages, phagocytosis of apoptotic neurons was delayed [133,134]. In addition to genetic factors, vascular risk factors for progressive dementia include atherosclerosis, hypertension, smoking, and diabetes [135].

Amyloid beta (Aβ)42/Aβ40 in plasma is a good tool to detect Aβ abnormalities in neurodegenerative diseases [136,137,138,139]. Neurofibrillary tangles are the two major histopathological markers of AD. Current lines of evidence revealed that destruction of the BBB in AD model mice was detected, and BBB permeability precedes senile plaque formation and cognitive deficits [140,141]. Another study demonstrated that NETs formed in the vessels of AD mice are one of the mechanisms of BBB disruption in AD pathology and progression [142] (Figure 3). Accumulated Aβ induces cerebral endothelial cells to express adhesion molecules such as intercellular cell adhesion molecule-1 (ICAM-1), and circulating neutrophils bind to ICAM-1 mediated by lymphocyte function-associated antigen-1 (LFA-1) [14]. In an AD mouse model [143], systemic inflammation preceded AD, including cognitive decline [38].

Neutrophil deletion in APP/presenilin-1 mice improved memory function and reduced microgliosis, leading to neuroinflammation [71]. The main causative factor within AD pathology is the beta-amyloid that activates microglia and attracts peripheral monocytes [144]. Circulating neutrophils of patients with AD contribute to neuronal and axonal damage and are one of the various pathophysiological mechanisms of AD. Aggregated neutrophils in the brain vessels impede cerebral blood flow. Blood plaques formed by thrombin and platelet aggregation block the blood flow in the microvessels. Hypoxia induces β-secretase activity and reduced Aβ clearance. [142]. During inflammation, the number of hyperactivated neutrophils increases and alters the CXCR4/CD62L ratio in fast-declining patients with cognitive dysfunction: CXCR is high and CD62L low [145]. Neutrophil CD11b is highly expressed in patients [146]. Infiltrating neutrophils from a mouse AD brain were detected as early as six months after the onset of aging and experimental AD symptoms occurred [71]. AD progression was hindered by the depletion of mature neutrophils, and infiltrating neutrophils and monocytes were also observed. Although the roles of Tregs and B cells remain controversial, the dominant response of Th1 and Th17 cells has been proposed as a hallmark of the immune response in AD [147,148]. It has been suggested that increased levels of TNF α and decreased levels of transforming growth factor β are observed in the CSF of patients with MCI [149]. Neuroinflammation in the pathological process of AD is caused by leukocytes such as lymphocytes, monocytes, and neutrophils [14,150,151]. NET-forming neutrophils are observed in the parenchyma and blood vessels of AD model mice and are involved in neutrophil-mediated chronic neuroinflammation, ultimately promoting the pathogenesis or development of AD. Activated neutrophils and imbalanced homeostasis correlate with accelerated cognitive decline. IL-17 originates from NETs and is released by LFA-1-dependent infiltration by neutrophils [145]. The binding of LFA-1 to ICAM-1 induces neutrophils to adhere to endothelial cells and infiltrate the brain parenchyma. Neutrophils regulating monocyte recruitment may be observed in inflammatory brain tissues at different time points during AD development.

### 4.4. Parkinson’s Disease (PD)-Derived Cognitive Impairment and Neutrophils

Neurodegenerative diseases with cognitive dysfunction, such as AD or PD, have been treated with disease-modifying therapies targeting the amyloid cascade and tauopathy. These pathologies are caused by neuronal death and loss of axonal connectivity. PD is a neurodegenerative disease characterized by the death of dopaminergic neurons in the substantia nigra of the midbrain [152]. Unlike AD, the progression of PD is related to chronic inflammation induced by glial activation and the recruitment of hematogenous immune cells [51]. Infiltrating monocytes and upregulated neutrophils produce MPO, affecting the midbrain subregion, and MPO-positive cells have been detected in the brains of patients with PD [153]. In an animal model of PD, MPO-deficiency deprived dopaminergic neurons, which have been implicated in the pathophysiology of PD [153].

The immune system of patients with PD is accelerated by aging and is easily epigenetically modulated in older adult patients than in age-matched controls [154]. Neutrophil levels increase in patients with PD, and the progression or severity of the disease is strongly correlated with peripheral neutrophil count [154]. Until now, there has been a potential connection between increasing immune cell counts, including neutrophil counts and activation, and age in PD. However, the development of suitable neutrophil modulation could be applied to immune therapies for PD and PD-derived dementia. A recent study demonstrated a relationship between peripheral inflammation, lymphocyte counts, NLR, and cognitive dysfunction in specific domains in patients with PD and MCI [155]. Evaluation of total lymphocyte counts and NLR in patients with PD with or without MCI revealed differences in the immunological assessment. The study provided accuracy as a biomarker of PD progression when blood lymphocytes or NLR and MCI were used for the prognosis of PD progression, particularly in cognitive impairment [155] (Figure 3). Patients with PD with MCI displayed significantly lower NLR and higher lymphocyte levels than those displayed in patients with PD without MCI. However, there were no differences in the erythrocyte sedimentation rate and C-reactive protein, which are used as indices for peripheral inflammation [155]. The authors explained that high levels of lymphocytes in patients with PD and severe cognitive decline could result from altered lymphocyte subpopulations.

Overall, this section discussed the potential role of neutrophils in cognitive impairment and neurodegenerative diseases. Neutrophils can interact with other inflammatory blood cells and regulate their function, potentially accelerating damage progression or severity. Additionally, neutrophils can destroy the blood–brain barrier, leading to subsequent neuronal damage. Neutrophil numbers and activation are altered in various neurodegenerative diseases and may be involved in disease progression and severity. With other factors contributing to cognitive impairment, such as genetic variants, vascular risk factors, and neurodegenerative markers, modulation of neutrophil activity may be a promising strategy for supporting neuronal regeneration. However, more research is needed to fully understand the relationship between neutrophils and cognition.

## 5. Neutrophil-Mediated Prevention for Progression of MCI to Dementia

In general, patients with MCI derived from various risk factors, including aging and cerebrovascular damage, are vulnerable to progression to dementia, particularly in patients with AD. Inflammation is an important pathological cause and contributor to the progression of AD. The current findings indicate that neutrophils derived from various neurodegenerative diseases are involved in MCI and its progression to dementia. Thus, specific neutrophil markers are required to predict the progression and prognosis of AD. The NLR relates to amyloid burden and cognitive decline in patients with AD [41]. A recent study indicated that neutrophil activation is associated with AD progression evaluated by the CDR and MMSE score change [145]. Another study reported that CD11b, a neutrophil adhesion molecule, correlated with deterioration of dementia assessed by daily living activities and a neuropsychological battery, and progression of cognitive impairment measured by the Token test [146]. These studies proposed that neutrophil markers in the peripheral blood may predict cognitive decline in patients with MCI in early AD [13]. Furthermore, specific markers for neutrophil activation related to the progression of cognitive decline in healthy participants, which could be applied to cognitive declining progression of the individuals as prognostic and diagnostic markers (Figure 3).

Pathogens are eliminated by neutrophils via phagocytosis by acting as phagosomes [34], releasing cytotoxic enzymes from neutrophil granules [35], trapping via NETs formed with ejected neutrophil DNA chromatin [36], and ultimately undergoing apoptosis such as cell death, NETosis [37]. When pathogen clearance is complete, most neutrophils undergo apoptosis, and pathogens are phagocytosed by macrophages [156]. Among the multiple granules and vesicles that release inflammatory factors, NGAL is released during degranulation to kill bacterial pathogens [157]. MPO is released from azurophilic granules, involves in cytotoxic ROS production [158], and eventually forms NETs [159]. In clinical studies, disease progression in AD has been associated with changes in neutrophil activity and ROS levels [58]. Postmortem studies support this relationship by reporting the presence of neutrophils in the vasculature of AD brains [57], co-localization of neutrophils with amyloid beta [14], and extensive NET formation in the brain vasculature of patients with AD [142]. Taken together, NGAL and MPO have emerged as candidate biomarkers, and other markers related to neutrophils have also been suggested. According to a meta-analysis of 35 studies, MPO and NGAL levels in the blood were significantly higher in patients with AD than in healthy controls (HC) [58]. In particular, NGAL concentrations in the peripheral blood were higher in patients with MCI than in HC. Intracellular plaques and neurofibrillary tangles in dementia are commonly associated with vascular injury [160]. In AD animal studies, neutrophils adhere to small cerebral vessels, and the inhibition of neutrophils enhances cerebral blood flow and improves memory performance [14]. Although peripheral neutrophil markers have been reported to predict executive functional decline in patients with MCI derived from cerebral small vessel disease, the roles of neutrophils within the CNS in AD were not mentioned, at least in peripheral blood findings [13].

Intravascular NETosis has been reported to play a role in atherothrombogenesis in patients with ischemic stroke internal carotid artery occlusion [161]. Anticoagulant drugs were introduced to target NETs, expecting a reduction in the risk of thrombosis and prevention of stroke attacks. Activation of neutrophil Nox promotes ROS overproduction in AD and induces NETosis in the brain parenchyma [117]. Several drugs have been developed to inhibit NET release and reduce NET formation in the CNS, including ticagrelor [162], colchicine [163], prostaglandins [164], and ruxolitinib [165]. However, when treating NET-associated CNS diseases, the problems to be solved are the permeability of the BBB and lesion specificity. In addition, serious immune system infections are caused by reduced NETs, unexpected side effects occur, and synergistic effects when combined with other traditional treatments may occur.

The roles of neutrophils and NETs in CNS diseases have been investigated and applied in basic and clinical research. Recently, several strategies have been developed to target neutrophil-driven pathologies. It has been implicated in neuroinflammation and progression of MCI to dementia in severe and progressive cognitive dysfunction. However, our understanding of the contribution of neutrophils to cognition under various conditions remains limited. Although neuroinflammation affects behavior and cognition, it is necessary to elucidate the role of neutrophils in this system. In this review, neutrophils are summarized based on earlier reports and latest research data. In the context of cognition, neutrophils are involved in the prevention of progression from MCI to dementia, particularly AD. During the process of eliminating pathogens through neutrophil-mediated mechanisms such as phagocytosis, the release of cytotoxic enzymes, and the formation of NETs, excessive neutrophil activity and the release of ROS can contribute to neuroinflammation. In AD, neutrophils have been found in the vasculature of the brain and are associated with amyloid beta and NET formation. Neutrophils and NETs have also been implicated in atherothrombogenesis and ischemic stroke. Inhibition of NET release is being explored as a potential therapeutic approach for reducing thrombosis and preventing stroke attacks. Overall, understanding and modulating neutrophil activity may have implications for inhibiting cognitive decline and neuroinflammation in various neurodegenerative diseases.

## 6. Autophagy-Mediated Neutrophil Regulation in MCI

One of the hallmarks of aging and degenerative diseases is the disruption of protein homeostasis and proteostasis, which is triggered by misfolded, mislocalized, and uncleared aggregated proteins. These abnormally aggregated proteins are cleared by the ubiquitin–proteasome system and autophagy that are central regulators of cellular proteostasis [166,167]. Autophagy is hampered by aging and accumulation of aggregated proteins. Disrupted autophagy triggers inflammation, a major repressor of age-related vessels in the brain [168]. Chronic inflammation develops progressively with age, termed inflammaging, and is implicated in various neurological disorders [169].

Autophagy plays an important role in regulating neutrophil function, and the regulatory role of neutrophils has clinical implications in CNS diseases such as cognitive impairment. In general, autophagy in neutrophils occurs at a basal level under nutrient-rich conditions and is induced by starvation, endoplasmic reticulum (ER) stress, oxidative stress, radiation, and hypoxia [170,171,172]. Autophagy regulates neutrophil-mediated inflammation, and controlled neutrophils affect their degranulation and ROS production via Nox [173]. Among neutrophil granules, primary granules (azurophilic) are formed in the ER–Golgi network and store MPO, β-glucuronidase, elastase, and other antimicrobial factors. Secondary granules are formed via both the ER–Golgi and endocytosis and include lactoferrin and MMP-9. Tertiary granules (gelatinases) are formed in the ER–Golgi network and release MMP-9. Granules play key roles in neutrophil migration and activation via complement factors and CD11b/CD18 [174]. Autophagy deficiency caused by ATG5 or ATG7 knockout inhibits neutrophils [175]. ER stress and neutrophil autophagy negatively affect each other during inflammation. ER stress leads to neutrophil autophagy, which is then inhibited by autophagy [175]. Similarly, autophagy downregulates ROS production and increases neutrophil count to control inflammation.

Vice versa, neutrophils also mediate autophagy, resulting in the regulation of neutrophil autophagy, degranulation, and NET formation. NET formation is an important step in the treatment of various stimuli [176] and comprises five stages: ROS generation, peptidyl arginine deiminase-4 activation, vesicle formation, chromatin decondensation, and extrusion of a peptide cocktail composed of DNA, histones, and cathelicidin [177]. In these stages, autophagy can interrupt NET vacuolization by externalizing membrane-bound and cytosolic proteins [178]. It has been reported that NET formation and autophagy are mediated by the phosphoinositide 3-kinase–protein kinase B–mTOR axis [179]. Autophagy decreases when mTOR is inactivated by dephosphorylation of Ser-2448. Autophagy inducers, such as rapamycin and WYE354, inhibit mTOR. In human neutrophils, treatment with autophagy inducers promotes NET formation via the formyl peptide receptor signaling pathway [180]. However, further evidence is needed to determine which component of autophagy is involved in NET formation, particularly in cognitive function and its progression to severe impairment and dementia. Neutrophil autophagy may be an effective target for treating cognitive impairment, in addition to various diseases, including inflammation, cancer, and infectious diseases.

The role of autophagy in regulating protein homeostasis and its implications in aging, neuroinflammation, and cognitive function are highlighted since disruptions in protein homeostasis, including the accumulation of misfolded proteins, are common features of aging and degenerative diseases. Autophagy is hampered by aging, leading to the accumulation of aggregated proteins and triggering inflammation, which is associated with age-related vascular issues in the brain. In the relationship between autophagy and neutrophils, neutrophils play a role in cognitive function, and their autophagy is regulated by factors like nutrient availability. Autophagy can also regulate neutrophil-mediated inflammation, and neutrophils, in turn, can modulate autophagy. It highlights the role of autophagy in interrupting neutrophil extracellular trap (NET) formation, a process that plays a critical role in immune responses to various stimuli. Taken together, modulating neutrophil autophagy could have clinical implications. Developing therapies that target neutrophil autophagy could potentially help manage inflammation-related cognitive decline. The intricate interplay between autophagy, neutrophils, inflammation, and cognitive function could provide potential avenues for clinical applications and further investigation into novel therapies for age-related cognitive impairment and related neurological disorders.

## 7. Conclusions

Evaluation of the progression rate from MCI to dementia, particularly in older adults, and predictions of the possibility or progressive stage in patients with MCI are becoming increasingly important, drawing the attention of researchers and clinicians. Aging is a decisive risk factor for neurodegenerative diseases and affects neutrophil count, phenotype, and function. This work discussed the clinical applications mediated by neutrophils and the direct and indirect effects derived from neutrophils in neurodegenerative diseases, including AD, PD, stroke, and atherosclerosis. Although neuroinflammation affects behavior and cognition, the exact contribution of neutrophils to cognition under various conditions remains limited. However, the currently available reports suggest that modulating neutrophils and their activity may be beneficial for inhibiting cognitive decline under different paradigms. One of these paradigms is the importance of autophagy-mediated neutrophil regulation in MCI, which is one of the hallmarks of aging and degenerative diseases. Autophagy is a cellular process that helps to maintain protein homeostasis and proteostasis, which is disrupted in aging and degenerative diseases. Therefore, further research is needed to elucidate the role of neutrophils in cognitive function and to develop strategies to target neutrophil-driven pathologies in neurodegenerative diseases. The role of neutrophils in autophagy-mediated regulation may provide new insights into the pathogenesis of neurodegenerative diseases and the development of novel therapeutic strategies.

## Data Availability

Not applicable.
